# Super‐Flexible Freestanding BiMnO_3_ Membranes with Stable Ferroelectricity and Ferromagnetism

**DOI:** 10.1002/advs.202102178

**Published:** 2021-10-28

**Authors:** Cai Jin, Yuanmin Zhu, Xiaowen Li, Feng An, Wenqiao Han, Qi Liu, Sixia Hu, Yanjiang Ji, Zedong Xu, Songbai Hu, Mao Ye, Gaokuo Zhong, Meng Gu, Lang Chen

**Affiliations:** ^1^ Department of Physics Southern University of Science and Technology Shenzhen 518055 China; ^2^ School of Physics Harbin Institute of Technology Harbin 150081 China; ^3^ Academy for Advanced Interdisciplinary Studies Southern University of Science and Technology Shenzhen 518055 China; ^4^ Department of Materials Science and Engineering Southern University of Science and Technology Shenzhen 518055 China; ^5^ Shenzhen Key Laboratory of Nanobiomechanics Shenzhen Institutes of Advanced Technology Chinese Academy of Sciences Shenzhen 518055 China; ^6^ Materials Characterization and Preparation Center Southern University of Science and Technology Shenzhen 518055 China

**Keywords:** ferroelectricity, ferromagnetism, freestanding BiMnO_3_ membranes, super‐flexible devices

## Abstract

Multiferroic materials with flexibility are expected to make great contributions to flexible electronic applications, such as sensors, memories, and wearable devices. In this work, super‐flexible freestanding BiMnO_3_ membranes with simultaneous ferroelectricity and ferromagnetism are synthesized using water‐soluble Sr_3_Al_2_O_6_ as the sacrificial buffer layer. The super‐flexibility of BiMnO_3_ membranes is demonstrated by undergoing an ≈180° folding during an in situ bending test, which is consistent with the results of first‐principles calculations. The piezoelectric signal under a bending radius of ≈500 µm confirms the stable existence of electric polarization in freestanding BiMnO_3_ membranes. Moreover, the stable ferromagnetism of freestanding BiMnO_3_ membranes is demonstrated after 100 times bending cycles with a bending radius of ≈2 mm. 5.1% uniaxial tensile strain is achieved in freestanding BiMnO_3_ membranes, and the piezoresponse force microscopy (PFM) phase retention behaviors confirm that the ferroelectricity of membranes can survive stably up to the strain of 1.7%. These super‐flexible membranes with stable ferroelectricity and ferromagnetism pave ways to the realizations of multifunctional flexible electronics.

## Graphical abstract

As flexible device have become vital in the forefront of technology, the maintenance of multifunctional performances under high flexion is highly desired. Here, the synthesis of super‐flexible freestanding BiMnO_3_ membranes with stable ferroelectricity and ferromagnetism simultaneously by pulsed laser deposition using recently promising water‐soluble Sr_3_Al_2_O_6_ as the sacrificial layer is reported.

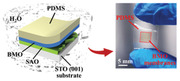

## 1 Introduction

Owing to their bendable and foldable capabilities, flexible materials have attracted considerable attention in recent years.^[^ ^1^, ^2^, ^3^ ^]^ Substantial efforts have been paid in wearable electronics, such as electronic skins,^[^ ^4^, ^5^ ^]^ wearable physiological monitoring devices,^[^ ^6^ ^]^ thin‐film transistors,^[^ ^7^ ^]^ and so on. However, achieving multifunctioning in materials of flexible form still remains a challenge. Recently emerging multiferroic oxide materials that show the potential applications of spintronic devices,^[^ ^8^ ^]^ photovoltaic devices,^[^ ^9^ ^]^ memories,^[^ ^10^ ^]^ and so on, provide an effective path for integrating properties for realizing the multifunction and flexibility of the materials simultaneously. BiMnO_3_ (BMO), as the sole strong ferromagnetic insulator (3.6 *μ*_B_ per Mn) in transition‐metal perovskite oxides,^[^ ^11^, ^12^, ^13^ ^]^ was reported recently to show ferroelectricity when subjected to epitaxial strain.^[^ ^14^, ^15^, ^16^, ^17^, ^18^, ^19^ ^]^ Being a rare multiferroic system with simultaneous ferroelectricity and ferromagnetism, the realization of flexibility in BMO is highly interesting in rewarding the development of future multifunctional devices.

Fortunately, recent novel peeling and transferring technologies for oxide thin films offer a viable solution to achieve the goal.^[^ ^20^, ^21^, ^22^, ^23^, ^24^, ^25^ ^]^ Using water‐soluble Sr_3_Al_2_O_6_ (SAO) as a sacrificial layer, ferroelectric BaTiO_3_ films were reported to be freestanding with super‐elasticity.^[^ ^26^ ^]^ In the same way, freestanding magnetic Fe_3_O_4_ membranes could also be bendable slightly.^[^ ^27^ ^]^ Here, we report the synthesis of flexible freestanding BMO membranes on soft supports. We reveal the freestanding BMO membranes with stable ferroelectricity and ferromagnetism simultaneously are super‐flexible with an ≈180° folding. First‐principles calculations were used to simulate the mechanical behaviors during the bending processes to further illustrate the super‐flexibility. The stable polarization and ferromagnetism of freestanding BMO membranes are demonstrated by the preserved performances after bending. Furthermore, we achieve 5.1% uniaxial tensile strain in freestanding BMO membranes and verify the ferroelectricity can survive stably up to the strain of 1.7%.^[^ ^17^ ^]^ Our work improves the possibility of achieving multiferroic functioning in flexible electronics.

## 2 Results and Discussion

**Figure 1:**
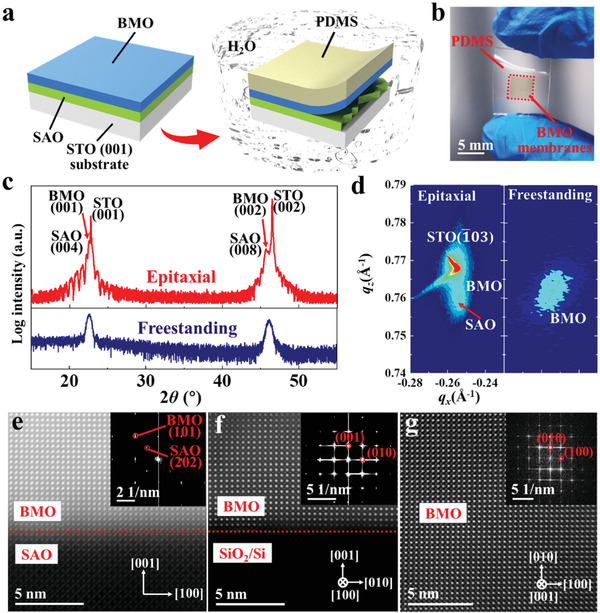
Synthesis and characterizations of the structure of freestanding BMO membranes. a) Schematic illustration of the as‐grown BMO/SAO heterostructures on STO substrates and the transfer process of dissolving the SAO sacrificial layer in water to form BMO membranes onto PDMS. b) Optical photograph of the flexible BMO membranes. c) XRD scans for epitaxial BMO/SAO heterostructures on STO substrates (top, red) and freestanding BMO membranes on PDMS (bottom, navy). d) Reciprocal space maps around the STO ($\bar{1}03$) peak for the epitaxial BMO/SAO heterostructures and the BMO ($\bar{1}03$) peak for freestanding BMO membranes. e) Cross‐sectional HAADF‐STEM image of BMO/SAO/STO heterostructure at BMO/SAO interface. The inset shows the corresponding FFT of the STEM image. f) Cross‐sectional HAADF‐STEM image of freestanding BMO membranes on SiO_2_/Si wafers. g) Plan‐view HAADF‐STEM image of freestanding BMO membranes. The inset shows the FFT image of the region from the STEM image, which reveals the pseudo‐cubic structure of the membranes.

BMO/SAO heterostructures were synthesized using pulsed laser deposition (PLD) on (001)‐oriented SrTiO_3_ (STO) substrates and then transferred to polydimethylsiloxane (PDMS) by dissolving the SAO layers via water (see **Figure** **1**a). Figure 1b shows the optical photograph of the resulting freestanding BMO membranes on PDMS, demonstrating the high flexibility. Only (001)‐type diffraction peaks were detected by X‐ray diffraction (XRD) *θ*–2*θ* scans, confirming the high crystallinity of BMO/SAO/STO heterostructures and freestanding BMO membranes (see Figure 1c). In addition, the only remained (001) reflections from BMO membranes demonstrate the complete dissolution of SAO sacrificial layers and the formation of high‐quality freestanding membranes. Reciprocal space mapping (RSM) around ($\bar{1}03$) diffraction peaks of STO and BMO is further used to confirm the quality of BMO/SAO/STO heterostructures and freestanding BMO membranes. As shown on the left in Figure 1d, the strain of SAO layers is released partially from the STO substrates with *a* lattices constant of 3.937 Å. The upper BMO films grew epitaxially on the SAO layers form the same in‐plane lattices of 3.937 Å, which is close to the reported value of *a*_pc − bulk,  BMO _(pc means pseudo‐cube) of 3.935 Å.^[^ ^12^ ^]^ Such close *a* value indicates that the BMO films grown on the SAO layers subject to minimal strain. Hence, as shown in the right in Figure 1d, the value of *a* and *c* lattices constant of transferred freestanding BMO membranes is basically the same as those of the BMO films grown on the SAO/STO layers. The morphology of as‐grown epitaxial BMO films and transferred freestanding BMO membranes measured by atomic force microscopy (AFM) is shown in Figure S1, Supporting Information. The consistent smooth surface illustrates the good topography retention after freestanding. In order to further identify the structures, we imaged the cross‐sectional scanning transmission electron microscopy (STEM) of epitaxial BMO films and freestanding BMO membranes. Figure S2a, Supporting Information, shows the low‐magnification STEM image of BMO (15 nm)/SAO (30 nm) heterostructure on (001) STO substrate. The corresponding energy‐dispersive spectroscopy (EDS) in Figure S2b, Supporting Information, shows the mapping of Al, Ti, Mn, Sr, and Pt elements, revealing sharp interfaces between homogenous BMO, SAO, and STO layers. Figure 1e is the expanded view of Figure S2a, Supporting Information, in the high‐angle annular dark‐field (HAADF) mode, indicating the high quality of BMO/SAO heterostructures. The inset shows the fast Fourier transform (FFT) of the STEM image, which reveals the pseudo‐cubic structure. The cross‐sectional atomic‐resolution HAADF‐STEM image of freestanding BMO membranes transferred on SiO_2_/Si wafers is shown in Figure 1f. The uniform atomic distribution with pseudo‐cubic structure (see the inset) indicates successful transfer processes. Furthermore, we also imaged the BMO membrane with plan‐view STEM. The corresponding expanded‐view HAADF‐STEM images of BMO membranes are shown in Figure 1g, indicating a stable structure of freestanding membranes after transferring. The large‐area HAADF‐STEM image and EDS mapping images of plan‐view freestanding BMO membranes shown in Figure S3, Supporting Information, also reveal the uniform distribution and high quality of freestanding BMO membranes.

**Figure 2:**
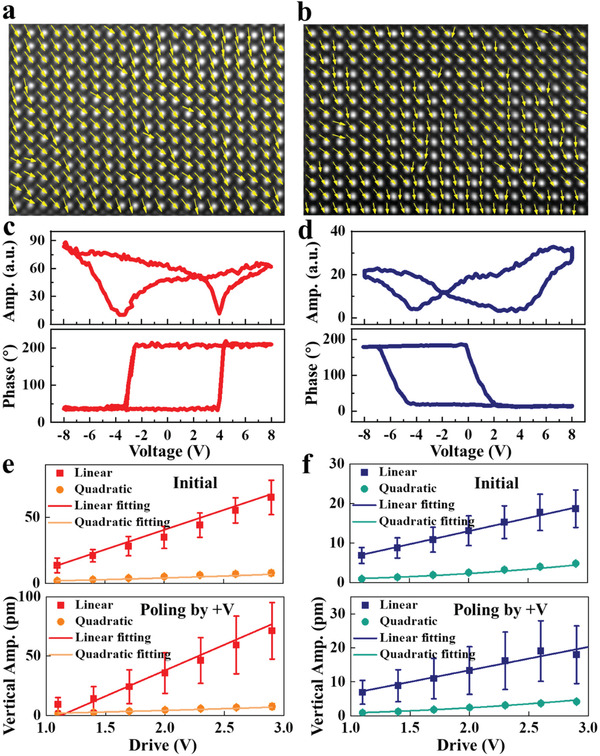
Polarized properties of as‐grown epitaxial BMO films and freestanding BMO membranes. a,b) Polarization vector mapping from “B”‐site atomic displacements in cross‐sectional HAADF‐STEM images of epitaxial BMO films and freestanding BMO membranes, respectively. The arrows indicate the direction of spontaneous polarization. c,d) Amplitude (Amp.) and phase of the PFM signal as a function of bias voltage for epitaxial BMO films and freestanding BMO membranes, respectively. e) The first and second harmonic piezoresponses of epitaxial BMO films measured at as‐grown regions (initial) and positive voltages poled regions (+V). f) The first and second harmonic piezoresponses of freestanding BMO membranes measured at initial regions and +V regions.

**Figure** **2**a,b shows the polarization vector mapping from “B”‐site atomic displacements in cross‐sectional HAADF‐STEM images of epitaxial BMO films and freestanding BMO membranes, respectively. As it shows, both the samples show the consistent direction of the arrows in the figures, presenting down‐polarization in the as‐grown states. The quantitative values of the “B”‐site atomic displacement of the STEM images along the in‐plane and out‐of‐plane directions are shown in Figure S4, Supporting Information. Both the epitaxial BMO films and freestanding BMO membranes display a certain offset from the center along the in‐plane and out‐of‐plane directions, which proves the existence of polarization in our samples. To identify the switching behaviors of the polarization, we performed piezoresponse force microscopy (PFM) measurements for our samples. Figure 2c,d shows the typical amplitude butterfly curves and phase hysteresis loops, reveal the switching behavior of BMO samples, indicating the stability of polarized states in our samples. Figure S5b,d, Supporting Information, shows the corresponding out‐of‐plane PFM poling map obtained in the selected regions where the smaller box inside was poled by +8 V DC voltage and the larger box inside was poled by −8 V DC voltage of epitaxial BMO films and freestanding BMO membranes, respectively. The distinct phase contrast between the square domain patterns reflects qualitatively the appearance of opposite polarizations, which indicates the strong piezoelectric responses of BMO samples. It should be noted that since the transferred BMO membranes are upside down on the PDMS, the freestanding BMO membranes present opposite polarization direction to the as‐grown films. The corresponding amplitude images in Figure S5a,c, Supporting Information, also show obvious domain walls. However, recent works report the differences in the contrast of the PFM amplitude and phase signals can also originate from artifacts.^[^ ^28^, ^29^, ^30^, ^31^ ^]^ To clarify this ambiguity, we examined the nature of observed local piezoresponses in detail by comparing the first and second harmonic responses averaged five spatial points in the initial regions and poled regions,^[^ ^31^, ^32^, ^33^ ^]^ as exhibited in Figure 2e,f and Figure S6, Supporting Information. It is observed that the first harmonic linear responses clearly dominate the second harmonic quadratic ones in all regions of both epitaxial BMO films and freestanding BMO membranes, suggesting the PFM responses measured are primarily piezoelectric and the contribution from charge injection is small. Combined with the previous reports of BMO films,^[^ ^14^, ^18^ ^]^ we consider above switching polarization in BMO probably stems from its intrinsic ferroelectricity.

**Figure 3:**
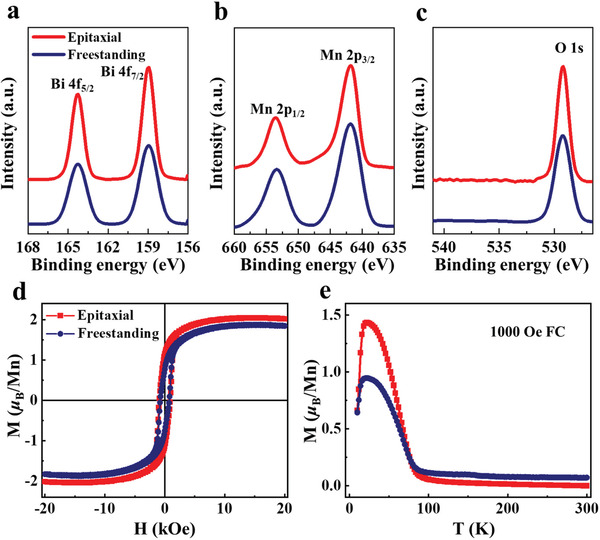
Basic characterizations including XPS spectra and magnetic properties of as‐grown epitaxial BMO films and freestanding BMO membranes. a) Bi 4f spectra, b) Mn 2p spectra, and c) O 1s spectra of the as‐grown BMO/SAO/STO epitaxial films (red) and freestanding BMO membranes (navy). d) In‐plane magnetic hysteresis (*M*–*H*) loops measured at 10 K. e) Temperature‐dependent magnetization (*M*–*T* ) curves of samples at 1000 Oe after field‐cooling.

We confirm the stable chemical composition during the transfer process by X‐ray photoelectron spectroscopy (XPS). As shown in **Figure** **3**a–c, neither the peak position nor the shape has changed in Bi 4f, Mn 2p, and O 1s spectrums after transferring. The Bi 4f_7/2_ and 4f_5/2_ are around 159.1 and 164.0 eV, respectively, originate from the Bi^3+^ ionic state in the crystal lattice.^[^ ^34^, ^35^ ^]^ Corresponding to Mn^3+^, the Mn 2p spectrum shows two main contributions (641.5 and 653.0 eV).^[^ ^36^ ^]^ Wherein, O 1s spectrum originates from the binding energy 529.5 eV, indicating O^2–^ state.^[^ ^36^ ^]^ Figure S7, Supporting Information, shows a survey scan of the XPS spectra of samples. Meanwhile, the magnetic properties of as‐grown epitaxial BMO films and freestanding BMO membranes are investigated in Figure 3d,e. Both epitaxial BMO films and freestanding BMO membranes exhibit consistent large in‐plane magnetic moment of about 2.0 *μ*_B_ per Mn with a *T*_C_ of ≈90 K, showing stable ferromagnetism in freestanding membranes. This ferromagnetic behavior found in BMO was reported to originate from the distortions imposed by the Bi lone pairs, leading to 3D ferromagnetic interactions of Mn^3+^‐O‐Mn^3+^.^[^ ^13^, ^17^, ^37^, ^38^ ^]^

**Figure 4:**
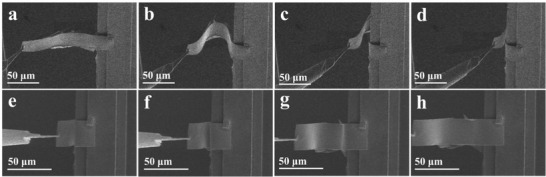
In situ SEM bending test of BMO nanobelts (160 µm by 30 µm by 120 nm). a–d) SEM images of BMO nanobelts under bending. e–h) SEM images of the recovery process.

To investigate the flexibility of freestanding BMO membranes, we transferred freestanding BMO membranes on the Cu grid and then fabricated freestanding BMO nanobelt using the focused ion beam (FIB) for in situ scanning electron microscopy (SEM) operations (see in Figure S8, Supporting Information). During the process, one end of the freestanding BMO nanobelt was fixed to the Cu grid using carbon coating, and the other end was welded with a nanomanipulator tip to push the BMO nanobelt to bend. As shown in **Figure** **4**a‐d, the flat BMO nanobelt was pushed by the attached nanomanipulator tip from left to right end, resulting in overall upward bending with an ≈180° folding. Intriguingly, as the fixed nanomanipulator tip was withdrawn from the right end, the BMO nanobelt recovered gradually to the initial flat state, indicating the mechanical stability of the freestanding BMO membranes with super‐flexibility (see Figure 4e‐h and Movie S1, Supporting Information). Recent works show that the ferroelectric domain plays a significant role in the super‐elasticity of freestanding membranes.^[^ ^26^, ^39^ ^]^ Likewise, the super‐flexibility of our BMO membranes most likely also stems from polarization domains to accommodate the flexure excitation for releasing strain.

Density functional theory calculations are also performed to obtain the mechanical properties of BMO nanobelt under bending deformations. During the bending processes of the BMO nanobelt, the upper surface is subjected to tensile strain and the lower surface is subjected to compressive strain (see Figure 4b and simulation of bending process in Movie S2, Supporting Information). According to the material mechanics theory, the bending curve *y*(*x*) of BMO nanobelt under bending strain satisfies the equation

(1) $$y\left(x\right)=y_{0}\sin^{2}\left(\frac{\pi x}{x_{0}}\right)$$

where *x*_0_ and *y*_0_ represent the maximum span and deflection distance, respectively. Therefore, the radius of curvature (*ρ*) can be expressed as

(2) $$\rho \left(x\right)=\frac{{\left(1+{y^{{}^{\prime }\left(x\right)}}^{2}\right)}^{\frac{3}{2}}}{y^{{}^{\prime \prime }\left(x\right)}}$$

**Figure 5:**
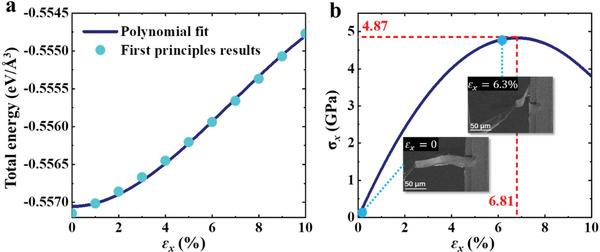
a) Total energy and b) tensile strain of the BMO nanobelt with the strain applied in the *x*‐direction (*ε_x_*) calculated by density functional theory.

In general, materials are more susceptible to damage by tensile strain, so we calculated based on the upper surface. We adopted tetragonal *P4mm* symmetry and fixed the lattice constant of BMO to the experimental value to simulate the BMO nanobelt under tensile strain. The relationship between the total energy *E*(ɛ_*x*_) and young's modulus under different uniaxial strains ɛ_*x*_ can be written as a Taylor expansion (see **Figure** **5**a)^[^ ^40^ ^]^

(3) $$E\left(\varepsilon_{x}\right)=E_{0}+\frac{1}{2}Y_{11}\varepsilon_{x}^{2}+\frac{1}{6}Y_{111}\varepsilon_{x}^{3}+\cdots$$

where *Y*_11_ and *Y*_111_ are the second‐order and third‐order Young's modulus, respectively. The uniaxial tensile strain *σ*_*x*_(*ε*_*x*_) is described by the gradient of the *E*(*ε*_*x*_)

(4) $$\sigma_{x}\left(\varepsilon_{x}\right)=\frac{\partial E\left(\varepsilon_{x}\right)}{\partial \varepsilon_{x}}=Y_{11}\varepsilon_{x}+\frac{1}{2}Y_{111}\varepsilon_{x}^{2}+\cdots$$

When the material reaches the ultimate strength

(5) $$\;\frac{\partial^{2}E\left(\varepsilon_{x}\right)}{\partial {\varepsilon_{x}}^{2}}=0$$

As shown in Figure 5b, the results of the first principles calculation show that the BMO nanobelt is able to subject to the *σ*_*x*_ of 6.8% and the *ε*_*x*_ of 5 GPa at the maximum, and the corresponding *ρ* is 882 nm. In our experiments, the *ρ* of BMO nanobelt reached a maximum of 950 nm, which was very close to the ultimate strength (see Figure 4d).

**Figure 6:**
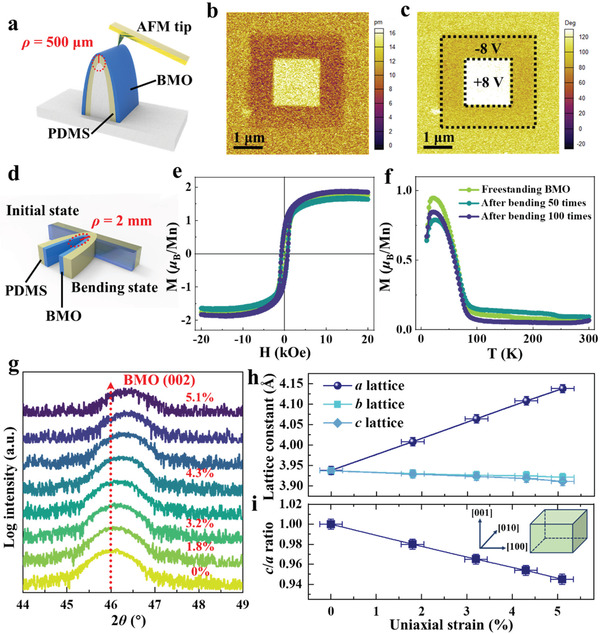
Physical properties of flexible BMO membranes under different states. a) Schematic of the bending stage for in situ PFM measurements with *ρ* ≈ 500 µm. b,c) Amplitude and phase images of the PFM poling map written at ± 8 V on freestanding BMO membranes. d) Schematic of bending state for magnetic measurements with *ρ* ≈ 2 mm. e,f) *M*–*H* loops and *M*–*T* curves of freestanding BMO membranes after undergoing different bending times, showing excellent durability. g) XRD scans around (002) diffraction peaks for freestanding BMO membranes with increasing uniaxial tensile strain, showing the decrease of the *c* lattice constant. h) Evolution of *a*, *b*, and *c* lattice constants of freestanding BMO membranes with the increasing uniaxial strain. i) Evolution of *c*/*a* ratio with the uniaxial strain. The inset shows the schematic diagram of the stretched state of the BMO unit cell.

Due to the outstanding super‐flexibility, we explore the physical properties of freestanding BMO membranes under extreme conditions. **Figure** **6**a shows the schematic of the bending stage with a *ρ* of ≈500 µm for PFM measurements. As shown in Figure 6b,c, the distinct contrast of phase and amplitude images of the PFM poling map written at ±8 V reveals the stable polarization of our freestanding BMO membranes. Also, comparing to the initial freestanding BMO membranes, the consistent in‐plane magnetic hysteresis (*M*–*H*) loops and temperature‐dependent magnetization (*M*–*T*) curves after undergoing 50 times and 100 times bending cycles with *ρ* of ≈ 2 mm, indicating the stable ferromagnetism of our freestanding BMO membranes with excellent mechanical durability (see Figure 6d‐f). Furthermore, we have integrated the freestanding BMO membranes onto a flexible polymer stretching platform to probe the tensile strain in BMO membranes.^[^ ^24^, ^25^ ^]^ The peaks shift at (002) diffraction measured by in situ XRD (see Figure 6g) indicates the consequent shrink of the *c* lattice constant as the uniaxial tensile strain was applied parallel to the [100] direction of the flexible freestanding BMO membranes. Meanwhile, the corresponding expansion of *a* and *b* lattice constant was detected by in situ RSM around the ($\bar{1}03$) and ($0\bar{1}3$) diffraction peak of BMO, respectively. The vertical red dashed lines (Figure S9a,b, Supporting Information) display the changes of *a* and *b* lattice constants as the uniaxial tensile strain was applied, respectively. And the horizontal red dashed lines show the changes of *c* lattice constants. Combining the above results, Figure 6h shows the final decrease of the *c* lattice constant from 3.937 to 3.912 Å, and the increase of *a* lattice constant from 3.936 to 4.138 Å. Moreover, only slight decrease of the other in‐plane lattice constant *b* is observed during the tensile tests. Such an expansion of *a* lattice means uniaxial tensile strain of 5.1% has been achieved on our freestanding BMO membranes, which has not been realized in epitaxial BMO films.^[^ ^17^ ^]^ PFM measurements of phase retention behaviors that reflect the ferroelectric properties were carried out to explore how stretched strain affects ferroelectricity in our BMO membranes.^[^ ^18^, ^41^, ^42^ ^]^ As it shows in Figure S10, Supporting Information, the membranes still display decent out‐of‐plane phase signals even after 30 min when the tensile strain is less than 1.7%, but weak as it increases gradually to 3.2%. Finally, the corresponding phase signal disappears completely under the tensile strain of 5.1%. Meanwhile, no in‐plane piezoelectric signal has been detected. Figure 6i shows a final decrease of *c*/*a* ratio to ≈0.945 of freestanding BMO membranes when uniaxial strain applies gradually until 5.1%. Among strained ferroelectrics, perovskite oxides with giant tetragonality (*c*/*a*) tend to have a large value of polarization and dipolar moment.^[^ ^43^, ^44^, ^45^ ^]^ In that sense, the disappearance of ferroelectricity of our BMO membranes in tensile tests is most likely due to the large decrease in the *c*/*a* ratio caused by the huge lattice change.

## 3 Conclusions

In summary, we have obtained high‐quality super‐flexible freestanding BMO membranes with stable ferroelectricity and ferromagnetism based on the single‐crystalline BMO/SAO heterostructures fabricated by PLD. By dissolving the water‐soluble SAO sacrificial layer, BMO membranes can be lifted off and transferred onto other substrates. The super‐flexibility of our freestanding BMO membranes is demonstrated by undergoing an ≈180° folding during an in situ bending test. First‐principles calculations simulate the mechanical properties of the bending processes, further illustrating the super‐flexibility of freestanding membranes. Meanwhile, electric polarization and ferromagnetism are confirmed to be well preserved under extreme conditions. Importantly, 5.1% of uniaxial tensile strain is achieved on our freestanding BMO membranes with ferroelectricity surviving stably up to the strain of 1.7%. Our present work may contribute to the application of multiferroic materials in multifunctional flexible devices.

## 4 Experimental Section

### Epitaxial Thin Films Deposition

Water‐soluble SAO layer (30 nm) was grown first on (001) STO single‐crystalline substrates by PLD with a 248 nm KrF excimer laser with a background oxygen pressure of 200 mTorr and substrates temperature (*T*_substrate_) at 800 °C. Then, *T*_substrate_ was cooled at 20 °C min^−1^ to 600 °C, followed by the deposition of BMO films (15 nm) under a 10 mTorr oxygen atmosphere. For the deposition of the SrRuO_3_ (SRO) bottom electrode, *T*_substrate_ was maintained at 750 °C under a 100 mTorr oxygen atmosphere. During the whole processes, the distance between target and substrate was fixed at 6 cm, and the targets were ablated at a fluence of 1.5 J cm^−2^ with a repetition rate of 2 Hz. After the depositions, the samples were cooled at 20 °C min^−1^ to room temperature (RT).

### Freestanding BMO Membranes Transfer

BMO/SAO/STO heterostructures with a surface covering PDMS support layers were heated for 10 min at 100 °C, ensuring tight interfaces. Then, PDMS/BMO/SAO/STO heterostructures were immersed in the deionized water to dissolve the sacrificial SAO layers, resulting in freestanding BMO membranes on PDMS support. Regarding the plan‐view STEM characterization and in situ SEM bending tests, a holey carbon Cu grid was used to support freestanding BMO membranes. Furthermore, for the cross‐sectional STEM characterizations, XPS measurements, and in situ stretching experiments of freestanding BMO membranes, 100 µm thick polypropylene carbonate (PPC) films were attached to heterostructures for dissolving. Then, the PPC‐coated BMO films were transferred onto SiO_2_/Si wafers or polyimide sheets. Finally, the PPC layers were decomposed completely by heat in O_2_ at 260 °C for 1 h, resulting in clean freestanding BMO membranes.

### Structural Characterization

XRD (Rigaku, SmartLab 9 kW, Cu K*α*, *λ* = 1.5413 Å) was used to characterize the structure and strain state of the samples. The microstructure of epitaxial BMO films and freestanding BMO membranes were analyzed by double‐aberration‐corrected transmission electron microscopy (ThermoFisher Themis Z G2 60–300) equipped with a super EDS detector operated at an accelerating voltage of 300 kV. All the cross‐sectional STEM samples were prepared by FIB (ThermoFisher Scientific (FEI) Helios 600i) with the in situ lift‐out technique. In situ SEM bending and recovery tests of freestanding BMO membranes were conducted by FIB.

### Ferroelectric Measurements

Metallic SRO layers of ≈20 nm thick were deposited on the bottom or the top of the BMO films to serve as the bottom electrode. The PFM characterizations were performed using a commercial scanning probe microscope (MFP‐3D, Asylum Research) at RT. PFM hysteresis loops and out‐of‐plane poling maps were recorded in the dual alternating current resonance tracing (DART) mode. The special first and second harmonic piezoresponses measurements were conducted statistically and computationally in single frequency mode by intentional programming under DART mode on atomic force microscopy (Asylum Cypher), to visualize and tell the intrinsic piezoresponse and other artifacts. To identify the atom position and extract the quantitative displacement vector of the cation ion, the HAADF‐STEM images were first filtered by FFT using a lattice mask and a low‐pass annular mask, and then the ion positions are determined by fitting them to 2D Gaussian peaks using homebrew Matlab scripts.^[^ ^46^, ^47^ ^]^ And the displacement vectors of “B”‐site ions were calculated as a vector from the center of mass of its four nearest “A”‐site cations to each “B”‐site cation.

### Magnetic Measurements

The chemical composition of epitaxial BMO films and freestanding BMO membranes were characterized by XPS (ThermoFisher SCIENTIFIC) with Al K*α* X‐ray source (*hν*  =  1486.8 eV). A Quantum Design MPMS3 was used to measure the magnetic properties of the samples.

### Density of Functional Theory Calculations

Based on the density of functional theory, all first‐principles calculations were implemented by the plane wave projector augmented wave method in the Vienna ab initio simulation package (VASP) code.^[^ ^48^ ^]^ The exchange‐correlation functional adopted the generalized gradient approximation of the Perdew−Burke−Ernzerhof functional. The BMO nanobelt was calculated by using 10×10×10 Monkhorst–Pack K‐point mesh. The energy cut‐off value was 500 eV, and the structures were completely relaxed until their atomic Hellmann–Feynman forces were less than 0.005 eV Å^−1^. The convergence criterion of energy in the self‐consistency process was 10^–6^ eV.

## Conflict of Interest

The authors declare no conflict of interest.

## Supporting information

Supporting Information

Supplemental Movie 1

Supplemental Movie 2

## Data Availability

The data that support the findings of this study are available from the corresponding author upon reasonable request.
