# Supplementary material for: Super‐Flexible Freestanding BiMnO3 Membranes with Stable Ferroelectricity and Ferromagnetism
Source: Adv Sci (Weinh). 2021 Oct 28;8(24):2102178. doi: 10.1002/advs.202102178 (PMC8693045; doi:10.1002/advs.202102178)
Supplement: Supplementary file 1 — Supporting Information [file ADVS-8-2102178-s001.pdf]

ADVANCED SCIENCE
Open Access

Supporting Information

for *Adv. Sci.*, DOI: 10.1002/advs.202102178

Super-flexible Freestanding $BiMnO_3$ Membranes with Stable Ferroelectricity and Ferromagnetism

*Cai Jin, Yuanmin Zhu, Xiaowen Li, Feng An, Wenqiao Han, Qi Liu, Sixia Hu, Yanjiang Ji, Zedong Xu, Songbai Hu, Mao Ye, Gaokuo Zhong, Meng Gu, and Lang Chen**

Supporting Information

Super-flexible Freestanding $BiMnO_3$ Membranes with Stable Ferroelectricity and Ferromagnetism

Cai Jin,[1,2] Yuanmin Zhu,[3,4] Xiaowen Li,[1] Feng An,[5] Wenqiao Han,[1] Qi Liu,[1] Sixia Hu,[6] Yanjiang Ji,[1] Zedong Xu,[1] Songbai Hu,[1] Mao Ye,[1] Gaokuo Zhong,[5] Meng Gu,[4] and Lang Chen*,[1,6]

[1]Department of Physics, Southern University of Science and Technology, Shenzhen 518055, China.

[2]School of Physics, Harbin Institute of Technology, Harbin 150081, China.

[3]Academy for Advanced Interdisciplinary Studies, Southern University of Science and Technology, Shenzhen 518055, China.

[4]Department of Materials Science and Engineering, Southern University of Science and Technology, Shenzhen 518055, China.

[5]Shenzhen Key Laboratory of Nanobiomechanics, Shenzhen Institutes of Advanced Technology, Chinese Academy of Sciences, Shenzhen 518055, China.

[6]Materials Characterization and Preparation Center, Southern University of Science and Technology, Shenzhen 518055, China.

*E-mail: chenlang@sustech.edu.cn

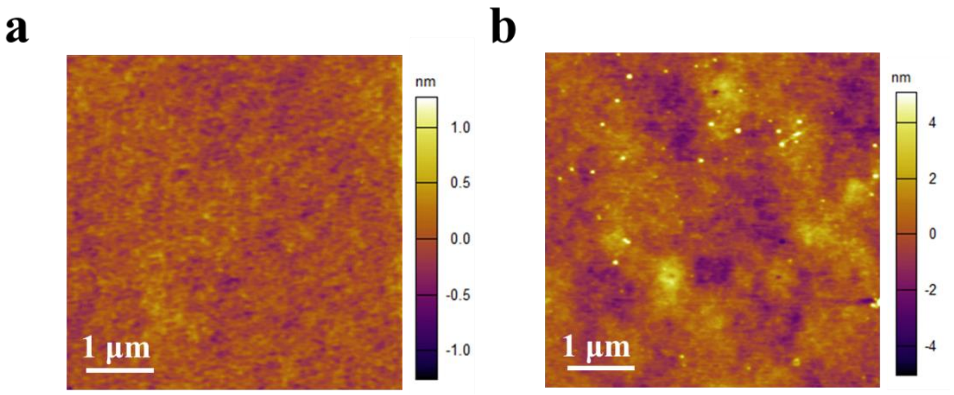

Figure S1. Morphology characterization of samples. a) and b) AFM images of as-grown BMO epitaxial films on SAO/STO and BMO membranes on PDMS, respectively.

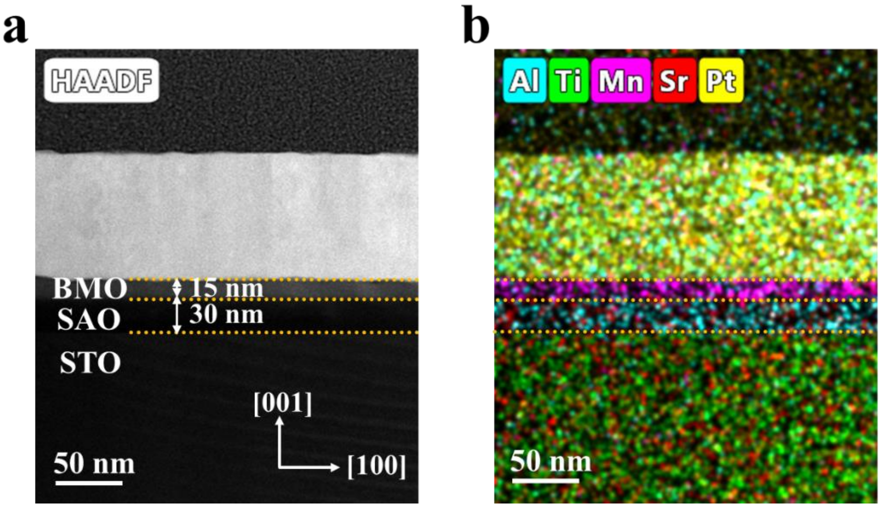

Figure S2. a) Low-magnification cross-sectional STEM image of BMO (15 nm) /SAO (30 nm) heterostructures on STO substrates. b) Corresponding EDS element mapping of as-grown BMO/SAO/STO epitaxial films, showing the Al, Ti, Mn, Sr and Pt elements, respectively.

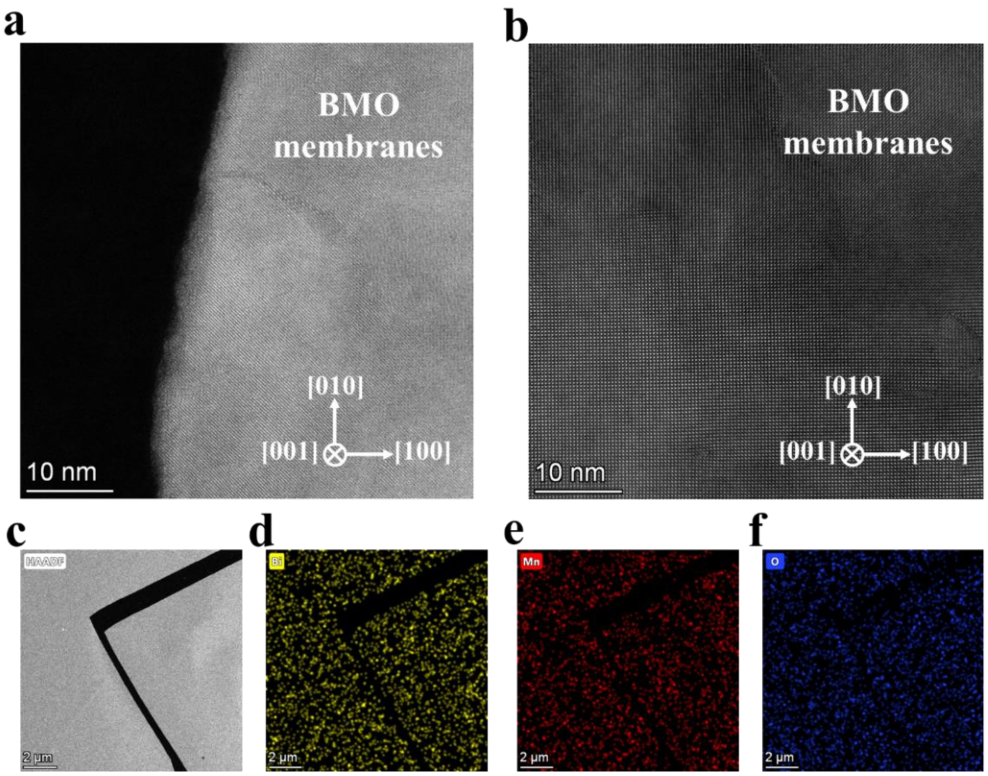

Figure S3. The microstructure of freestanding BMO membranes. a) Plan-view low-magnification STEM image of 15 nm-thick BMO membranes on a holey carbon Cu grid. b) Large area of plan-view HAADF-STEM image of freestanding BMO membranes. c)-f) Low-magnification selected area STEM image and corresponding EDS element mapping, showing the Bi, Mn and O elements, respectively.

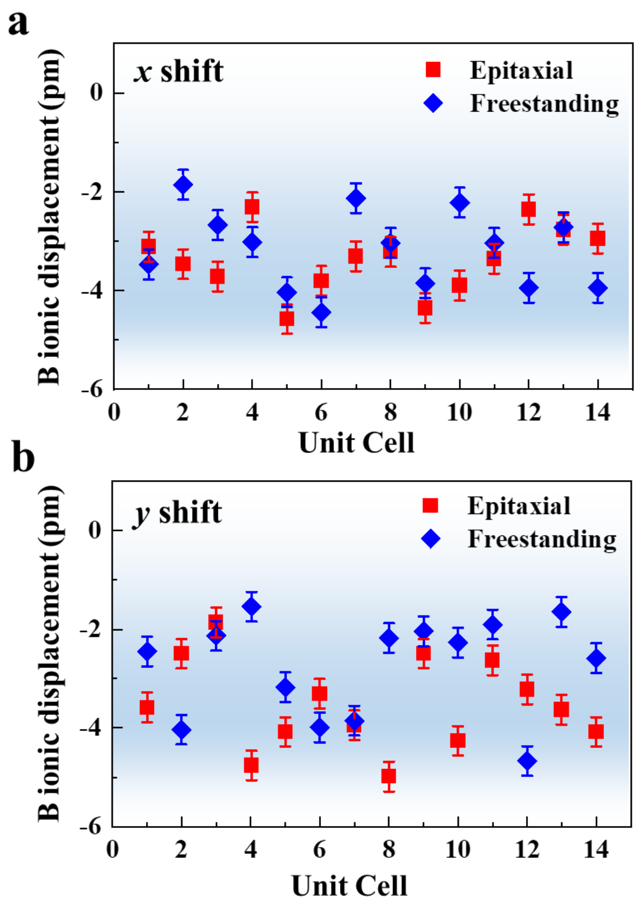

Figure S4. a) and b) The quantitative values of the "B"-site atomic displacement of the STEM images along the in-plane (*x*) and out-of-plane (*y*) directions.

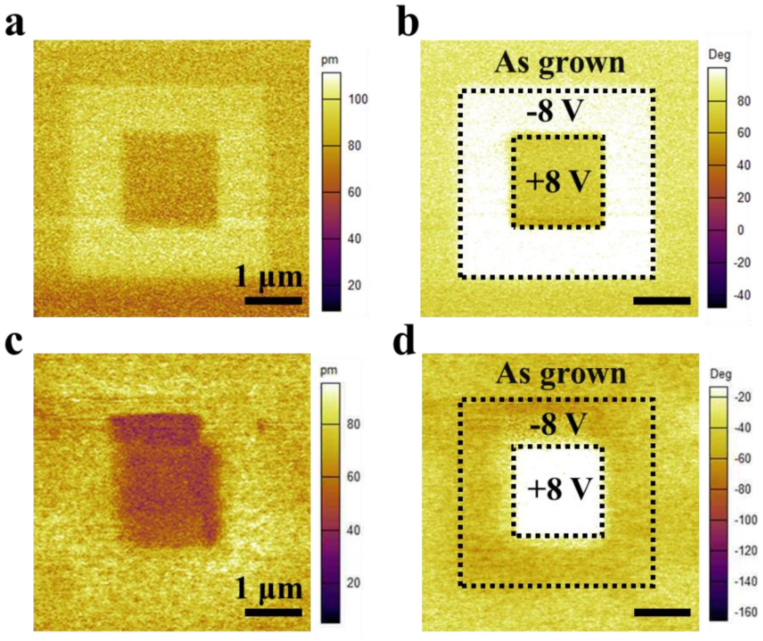

Figure S5. a) and b) Amplitude and phase images of out-of-plane PFM poling map written at ±8 V of as-grown epitaxial BMO films on SRO/SAO/STO, respectively. c) and d) Amplitude and phase images of out-of-plane PFM poling map written at ±8 V of freestanding BMO/SRO membranes on PDMS, respectively.

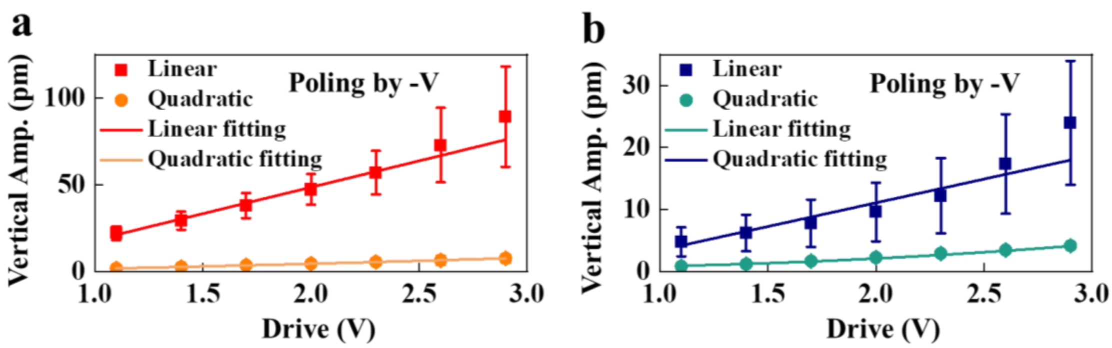

Figure S6. a) and b) The first and second harmonic piezoresponses of epitaxial BMO films and freestanding BMO membranes in the regions poled by negative voltages (-V), respectively.

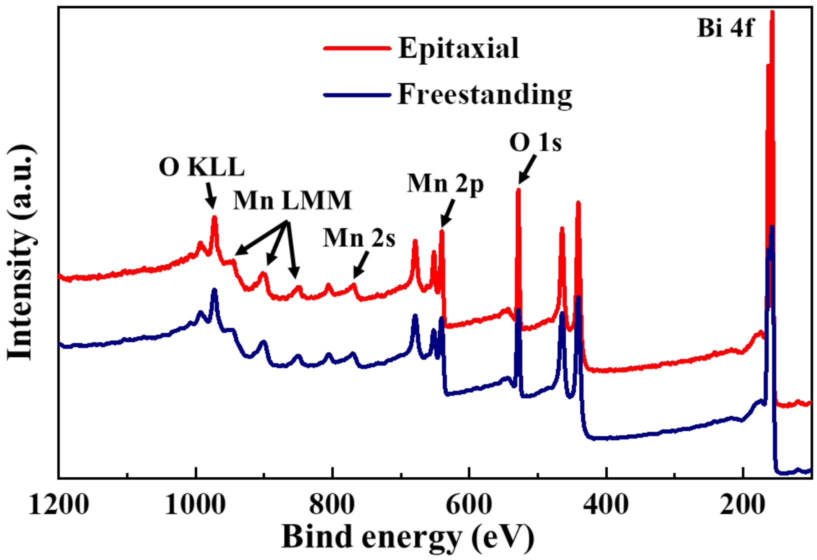

Figure S7. The wide scan XPS spectra of as-grown BMO epitaxial films (red) and freestanding BMO membranes on PDMS (navy).

a b

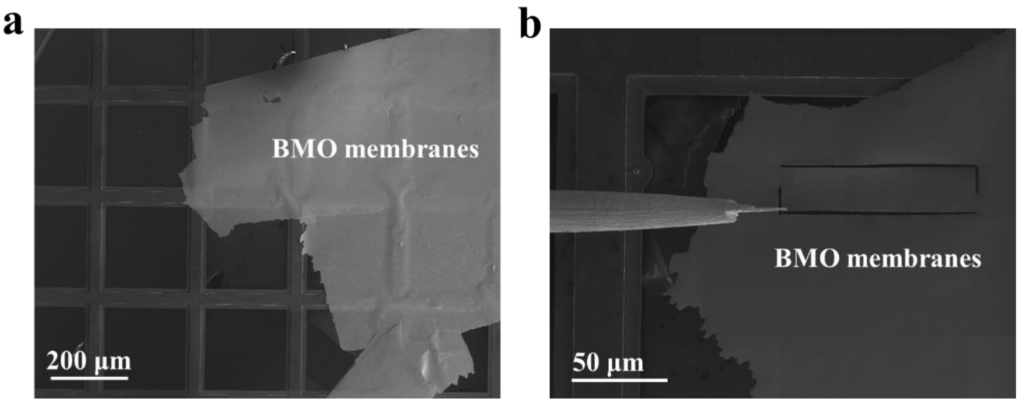

Figure S8. Preparation of freestanding BMO nanobelt. a) SEM image of freestanding BMO membranes (120 nm). b) SEM images of freestanding BMO nanobelt fabricated by FIB and handled by the nano-manipulator tip.

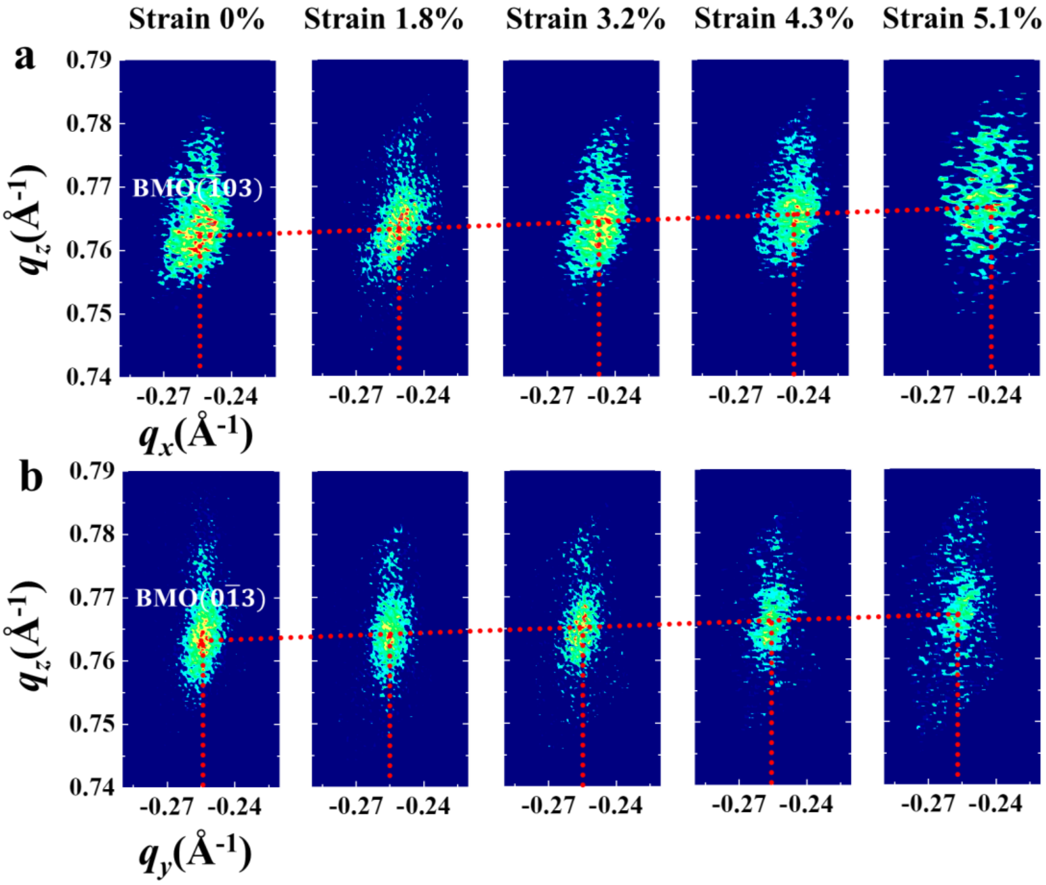

Figure S9. Evolution of *a* and *b* lattice constants of freestanding BMO membranes with the increasing uniaxial strain.

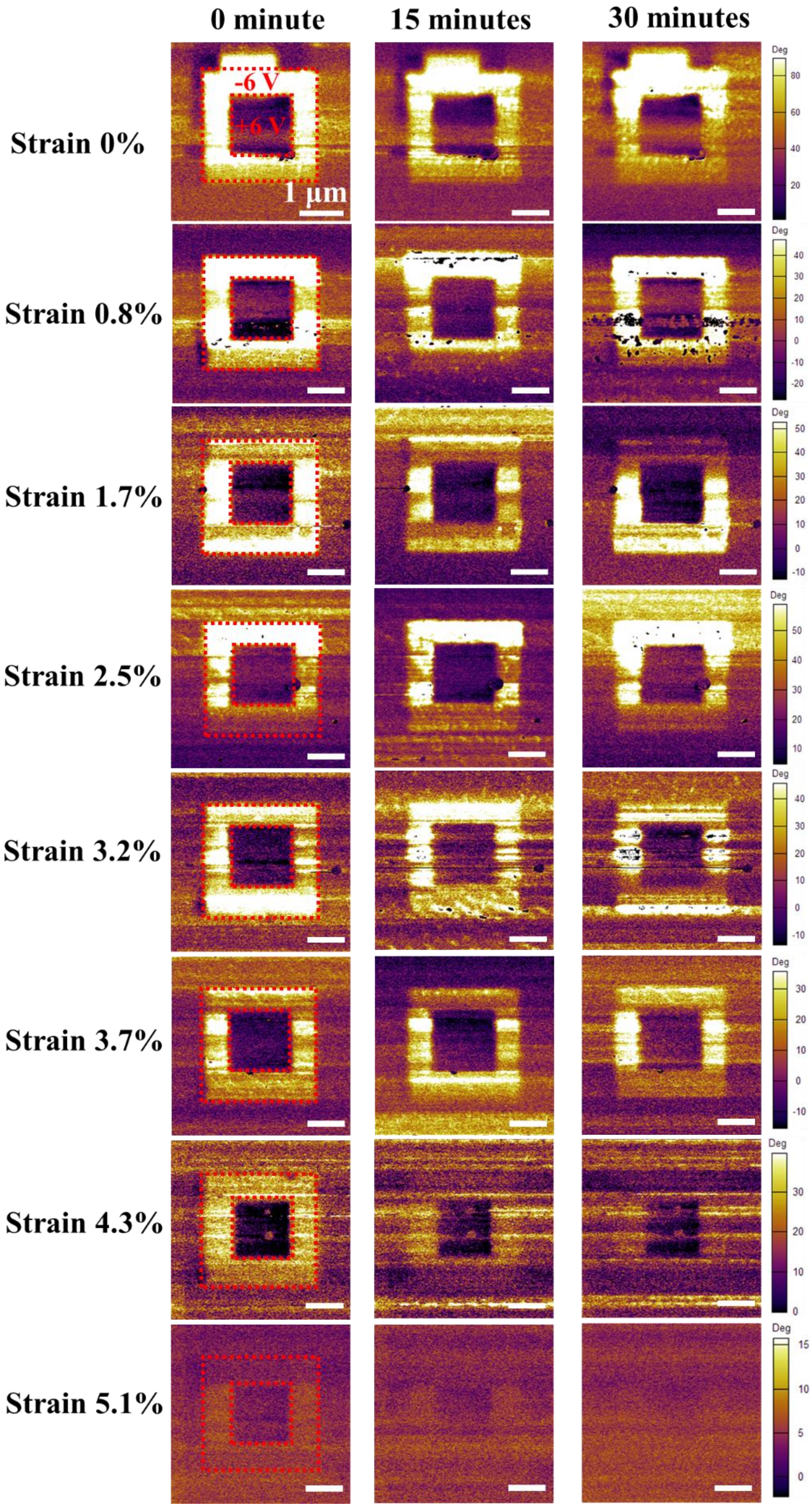

Figure S10. Ferroelectric retention behavior for freestanding BMO membranes with increasing uniaxial tensile strain. PFM phase images after the poling for different time intervals: 0, 15, and 30 minutes, respectively.
